# Oral function in patients with myasthenia gravis

**DOI:** 10.7717/peerj.11680

**Published:** 2021-06-29

**Authors:** Agnete Overgaard Donskov, Akiko Shimada, Lotte Vinge, Peter Svensson, Henning Andersen

**Affiliations:** 1 Department of Neurology, Aarhus University Hospital, Aarhus, Region midt, Denmark; 2 Department of Geriatric Dentistry, Osaka Dental University, Osaka, Japan; 3 Section of Orofacial Pain and Jaw Function, Department of Dentistry and Oral Health, Aarhus University, Denmark; 4 Scandinavian Center for Orofacial Neurosciences (SCON), Aarhus, Denmark; 5 Faculty of Odontology, MalmøUniversity, Sweden

**Keywords:** Myasthenia Gravis, Mastication, Oral function

## Abstract

Myasthenia Gravis (MG) is characterised by muscle weakness and increased fatigability. The aim of this pilot study was to investigate if patients with MG demonstrate different functional chewing patterns and report more complaints related to mastication as compared with healthy controls. Twelve patients (median 60 years Q1–Q3: 46–70) with generalised MG and nine healthy controls (median 57 years Q1–Q3: 55–63) participated. All participants underwent dental and oral examination and were asked to fill in a questionnaire concerning oral health. Static maximum bite force was measured with a bite force transducer, electromyography in the masseter, temporalis, and suprahyoid muscles were recorded, and jaw movement was tracked, during a 5-minute gum chewing test. The patients had more oral complaints (oral health impact profile total score 22.6 vs 7.5 *P* < 0.01) and had lower peak bite force than controls (18.8kgf (11.1;26.4) (95% CI) vs 29.5 kgf (21.6; 37.4) (*P* = 0.04)). In contrast, fatigability of the masticatory muscles, as defined by number of chewing cycles during the gum-chewing test, did not differ between patients and controls (*P* = 0.10). In conclusion, patients had more oral complaints and lower bite force than controls, but did not show significantly different functional chewing patterns. Future studies should aim at integrating measurement of peak force into functional tests. Attention should be given to oral complaints of patients with MG.

## Introduction

Myasthenia Gravis (MG) is an autoimmune disease at the neuromuscular junction where autoantibodies against the postsynaptic membrane molecules result in impaired neuromuscular transmission (Gilhus, 2016; Gwathmey & Burns, 2015; Conti-Fine, Milani & Kaminski, 2006; Tuzun & Christadoss, 2013). The antibodies target either acetylcholine receptors or related functionally molecules (LRP4 and muscle specific kinase) (Gilhus, 2016). The main clinical characteristic in MG is fluctuating muscle weakness with increased fatigue during continuous muscle activity (Gwathmey & Burns, 2015). The prevalence of MG is reported to be 140–150 in one million patients and the incidence ranges between 1.7 and 30 per million per year (Gwathmey & Burns, 2015; Querol & Illa, 2013; Heldal et al., 2009)

In MG, bulbar symptoms include problems related to chewing, swallowing and talking (Weijnen et al., 2002). Although chewing is rarely an initial symptom, it is a frequent patient complaint (Gwathmey & Burns, 2015; Sasakura et al., 2000). Problems occur during closure of the jaw caused by impairment of the masseter muscles (Pal & Sanyal, 2011). Combined with tongue weakness (Weijnen et al., 1998), this may cause difficulties when eating leading to malnutrition in severe cases (Weijnen et al., 2002; Keesey, 2004). Anti-myasthenic treatment aims at improving quality of life and level of physical function (Gilhus, 2016) including oral functions. Studies are lacking on the impact of the chewing on oral health.

The aim of this pilot study was to evaluate complaints of oral function and to quantify the strength of chewing muscles in patients with MG, compared with healthy controls. We hypothesised that patients with MG report more oral complaints and have a lower bite force, increased muscle fatigability and impaired functional chewing compared with healthy controls.

## Material and methods

### Participants

Patients with MG were recruited from a cross-sectional study of all MG patients living in the Central Denmark Region, one of five Danish regions (Vinge, Jakobsen & Andersen, 2019). Inclusion criterion was generalised MG irrespective of the presence of bulbar symptoms. Exclusion criteria were pure ocular manifestations, jaw muscle pain and painful temporomandibular joint disorders. Before entering the study, eligible participants underwent an examination of their teeth and oral cavity by a dentist. The clinical examination included the “Research Diagnostic Criteria for Temporomandibular Disorders” (RDC/TMD) to detect and evaluate any painful temporomandibular joint dysfunction (TMD) such as myalgia or arthralgia lead to exclusion of the participant from the study.

Twelve sex- and age-matched participants were included as controls,. Exclusion criteria for controls were jaw muscle pain and painful TMD. The severity of myasthenia in patients was evaluated using the clinical neurological evaluation scales MG Composite (Burns, 2012b), and Quantitative Myasthenia Gravis (QMG) (Barohn et al., 1998). The study was approved by the Central Denmark Region Committee on Health Research Ethics, registered at the Danish Data Protection Agency and given the number 1-10-72-12-13. All participants gave written informed consent before entering the study.

### Study design

All examinations were performed at Department of Dentistry and Oral Health, Aarhus University including clinical evaluations combined with self-reported and objective measures of mastication.

Participants were asked to fill in an Oral Health Impact Profile (OHIP) concerning perception of own oral health and its impact on quality of life. The OHIP contains 49 questions concerning oral function in chewing, eating, talking, as well as oral pain and discomfort (Gabardo, Moyses & Moyses, 2013). Answers are rated from 0-4 according to the amount of time the participant experienced the problem or discomfort in question from *“never / don’t know” (0), “almost never” (1), “sometimes” (2), “often” (3),* and *“very often” (4).* The total score ranges from 0 to 196.

The maximum bite force was measured using a U-shaped bite force transducer displaying force ranging from 0 –1000 (Kgf). The participants were asked to clench their teeth for 5 s and the highest bite force displayed was noted as their peak value. The test was repeated four times (Svensson & Arendt-Nielsen, 1996).

Maximum bite force was measured initially in the session and repeated after each of the following functional tests. After the static and functional tests the participants were given a two-minute rest before the next test was commenced. A bite force transducer provided the result of measured maximum bite force directly on the A/D converter display expressed in kgf as described by Svensson & Arendt-Nielsen (1996).

Electromyography (EMG) measurements were performed with bipolar EMG surface electrodes placed along the central part of the masseter muscle, the anterior temporalis muscles, and the suprahyoid muscles. The placement of the electrodes was made by palpation of the electrodes during maximum contraction. The Amplification of the EMG data was differentiated, filtered by 20–200 Hz and sampled at 512 Hz (Svensson, Arendt-Nielsen & Houe, 1996).

In one of the functional tests, participants were asked to chew test food for a maximum of one minute or until the food was swallowed completely. In the soft and brittle food test, a standardised cookie (half an “*Oreo”* mini biscuit without the cream) was used. In the non-brittle and hard food test, a piece of carrot was used measuring 25 mm in diameter, cut in squares and with a thickness of five mm. Finally, to evaluate muscle fatigue, the participants were asked to chew a chewing gum for five minutes. The number of chewing cycles was recorded with EMG and electrognathography (EGG).

During the functional tests, jaw movements were recorded at the lower incisor in the anterior, lateral and anterior-posterior axis with a sirognatograph (Svensson, Arendt-Nielsen & Houe, 1996). Participants were placed in upright position on a chair during measurements, and the recordings were stored as EGG signals.

### Analysis

The EGG recordings determined the onset of the opening of the jaw by change in the vertical axis, recorded in a 20-sample window. This synchronised the EGG and EMG data into time-adjusted single masticatory cycles. The rectified EMG waves were analysed and converted into numerical data using custom-made software “Oroface 2.1”. The number of chewing cycles in the fatigue tests was determined from the EMG-measured cycles of chewing. The static parameters of bite force were obtained directly from the bite force transducer.

### Statistical evaluations

Number of masticatory cycles, peak bite force and OHIP data were found to fit a normal distribution using the Kolmogorov–Smirnov test. The test for statistical significance between patients and controls was performed using a two-tailed *t*-test for unpaired data with unpaired variance. To calculate the most accurate confidence intervals for a limited data set the degrees of freedom were taken in account. A *P* value <0.05 was considered statistically significant. This test was used for testing the difference in responses in the OHIP questionnaire.

When testing for correlations, Spearman’s rank correlation test was used to test for significance in the correlation between peak-bite force and different clinical assessment scores in both patients and controls and the patient group alone.

## Results

### Participants

Fourteen patients with MG and 12 healthy controls were enrolled. Due to poor quality of five test recordings (two MG patients and three controls), only data from 21 participants were included in the analyses.

The final sample consisted of 12 sets of patient data and nine sets of control data, and a slightly skewed distribution of sex between the groups, with 66% women in the patient group and 55% women in the control group. In the patient group, the median age was 60 years (min-max:43–76 years) and 57 years in the control group (min-max:54–70 years). The RDC/TMD evaluation did not lead to exclusion of any of the 21 participants due to jaw muscle pain and temporomandibular joint problems.

### Clinical characteristics

All patients had generalised MG and all patients, except one received pyridostigmine (Mestinon^®^). Nine patients were treated with one or two additional drugs. The average MG composite score (±SD) was 10.9 ± 4.9, and the QMG score (±SD) was 8.4 ± 5.3.

### OHIP questionnaire

As shown in Table 1, the patients had higher scores than controls in the categories function and pain, as well as in the average sum of all categories in the OHIP questionnaire. In the category diet, there was no statistical significant difference between patients and controls (*P* = 0.05).

**Table 1:** Oral health impact profile and peak bite force in patients and controls. OHIP data and Averaged peak force (SD) for patients and controls. The average OHIP answer in each category can range from 0-4 and the total total sum can range between 0-196.

	Patients (*N* = 12)	Controls (*N* = 9)
**OHIP data**		
Diet	2.2	0.1
Function	5.3^*^	2.2^*^
Pain	7.4^*^	3.2^*^
Appearance and dentures	0.6	0.2
Mentally	4	1
Socially	1.2	0.7
Total score	22.6^**^	7.5^**^
**Peak force (kgf)**	18.8(12.1)^*^	29.5(10.3)^*^

**Notes.**

* *P* value < 0,05.

** *P* value <0.

### Static peak bite force

Patients had a lower peak bite force than controls (*P* = 0.04) (Table 1).

### Functional tests - hard and soft food

**Figure 1:**
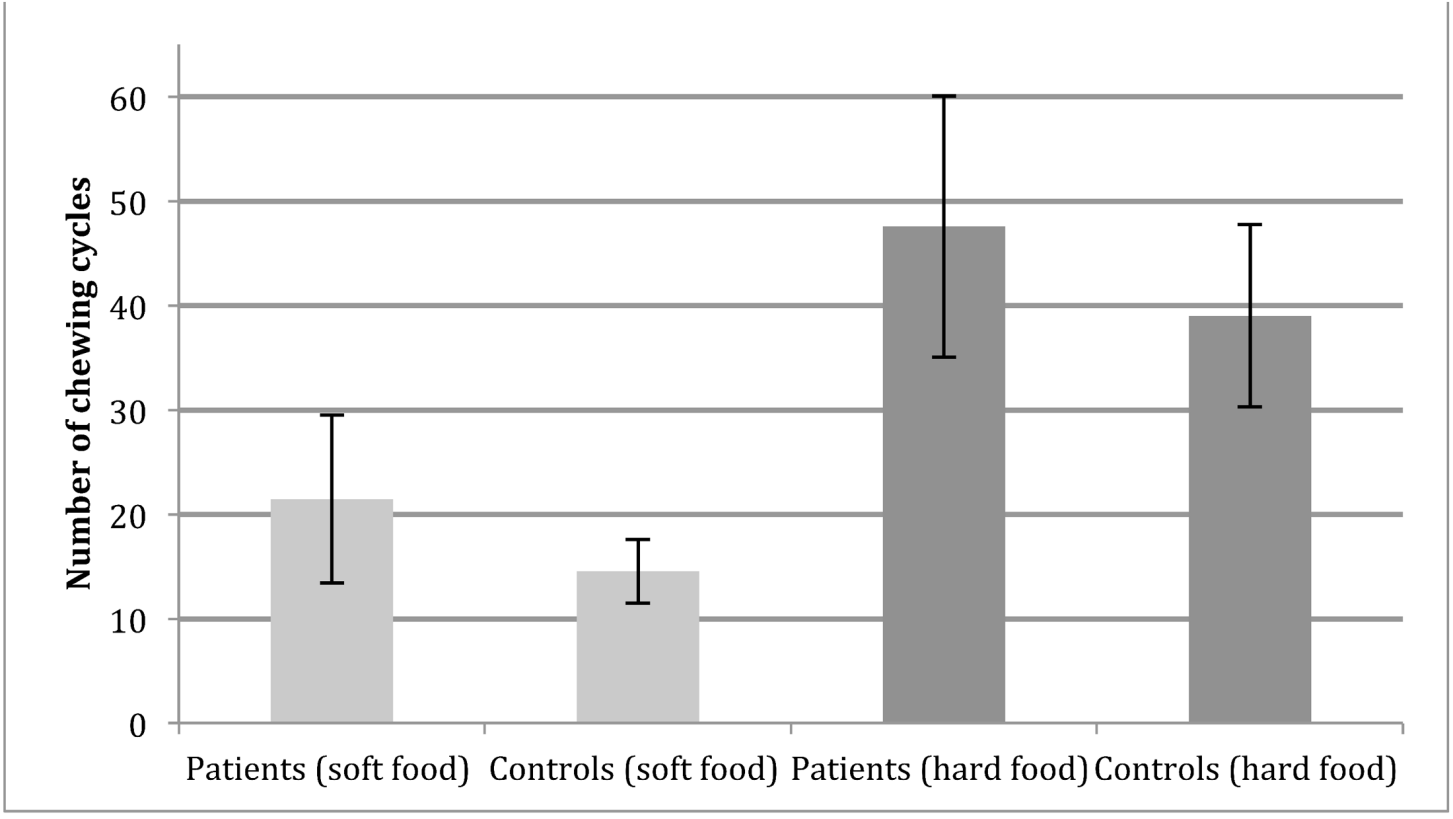
Functional chewing tests. Number of chewing cycles in patients (*N* = 12) and controls (*N* = 9) during functional testing, error bars shows the 95%CI calculated with eight degrees of freedom for control group and 11 degrees of freedom for patients. No significant difference between groups, as well as foods (*P* = 0.09 and *P* = 0.22 respectively).

Patients and controls did not differ when comparing the average number of chewing cycles needed for chewing and swallowing (Fig. 1) neither in soft (patients 21.4 and controls 14.5 (*P* = 0.09)) or hard food (Controls 37.0 and patients 49.5(*P* = 0.22)). One patient was unable to finish the mastication of the carrot in one minute in all of the three, repeated hard food mastication tests.

### Fatigue test

In the fatigue test, the continuous chewing for five minutes on chewing gum was recorded quantitatively to investigate if there was any difference between the patients and controls in number of masticatory cycles. There was no significant difference in number of chewing cycles between the groups (control group (391 cycles) patient group (338 cycles) (*P* = 0.10)).

### Correlations

To evaluate the correlation between disease severity and the maximum bite force, a correlation analysis was performed in the patient group between the maximum bite force and the measures of clinical severity of MG (QMG and MG composite). No parameter correlated significantly with the peak bite force including QMG (r = −0.26 (*P* = 0.43)).

## Discussion

The two main findings in this pilot were (1) a lower oral health reported by the patients with MG, and (2) a lower peak bite force in patients with MG. Patients reported more complaints concerning oral function and its effect on quality of life compared with controls. Although disease control and average life span are often achieved with anti-myasthenic treatment (Gilhus, 2016) MG is a chronic, fluctuating disease known to impact on patients’ quality of life (Burns, 2012a). Our study calls for larger investigations to further clarify the impact of oral health on quality of life in MG.

Two out of 12 patients received Methotrexate and four out of 12 patients received prednisolone in addition to Mestinon^®^ treatment. Both drugs are known to increase the risk for mucosal lesions and *Candida* (Pedrazas, Azevedo & Torres, 2010), and cause candidiasis and loss of bone density in the mandibular bone (Beeraka et al., 2013). It is thus relevant to consider the association between oral complaints and long-term treatment with both prednisolone and methotrexate treatment to evaluate any possible adverse effects.

We found that patients with MG had a lower peak bite force than healthy controls. Reduced peak force has been reported in previous studies of masticatory function in patients with MG (Weijnen et al., 2002; Weijnen et al., 1998; Weijnen et al., 2000; Bilt et al., 2001) and has been suggested (Tuzun & Christadoss, 2013) to be caused by irreversible damage to the postsynaptic membrane at the neuromuscular junction. In line with our findings, no previous studies (Weijnen et al., 2002; Bilt et al., 2001) using EMG amplitude measurements have enabled detection of increased fatigability in patients with MG. Even when performed a five-minutes fatigue test (Bilt et al., 2001), we did not find an increased fatigability in patients compared with controls. Our findings of lower muscle strength without fatigue are in line with our previous study of extremity muscles in MG (Vinge, Jakobsen & Andersen, 2019).

### Methodological limitations

The present study design aimed at measuring both static and dynamic-functional mastication performance. Elsewhere, EMG has been used to measure peak force and correlate this to peak bite force (Weijnen et al., 2002; Bilt et al., 2001). As EMG is a proxy measure, we chose to quantify the functional bite force output directly in kgf, comparable and convertible to Nm. However, recording EMG during both maximum bite force and in functional tests, allows evaluation of the relative muscle activity usage in functional chewing. Previous studies (Weijnen et al., 1998; Weijnen et al., 2000) have used this approach to compare of muscle activity and muscle function as measured by kgf or Nm. Furthermore, it would be relevant to include a measurement of peak force during the functional tests of mastication, a technique that has been proved possible (Shimada et al., 2012).

Additionally, since performing the tests, the RDC/TMD has been updated to the DC/TMD (Schiffman et al., 2014). Thus in future studies these diagnostic criteria should be applied.

## Conclusion

Patients with MG have both lower self-reported oral health and lower bite force. In the functional chewing-tests, fatigability was not increased in patients with MG compared to controls. Future studies should aim at integrating measurement of peak force into the functional tests to evaluate the relative force expenditure of functional chewing in patients with MG as compared to their peak force performance. Since the aim of myastenic treatment is improved quality of all aspects of life, oral health should also be evaluated. We thus suggest applying the OHIP questionnaire in future studies of MG mastication.

##  Supplemental Information

10.7717/peerj.11680/supp-1 Supplemental Information 1 EMG controls

10.7717/peerj.11680/supp-2 Supplemental Information 2 Peak force values

10.7717/peerj.11680/supp-3 Supplemental Information 3 EMG data

10.7717/peerj.11680/supp-4 Supplemental Information 4 Number of Mastication cycles Patient and control data of masticatory cycles and peak bite force values.

10.7717/peerj.11680/supp-5 Supplemental Information 5 Questionnaire in Danish

10.7717/peerj.11680/supp-6 Supplemental Information 6 Questionnaire in English

## Abbreviations

MG: Myasthenia Gravis
OHIP: Oral Health Impact Profile
QMG: Quantitative Myasthenia Gravis
MGC: Myasthenia Gravis Composite
RDC/TMD: Research Diagnostic Criteria for Temporomandibular Disorders
EMG: Electromyography
EGG: Electrognathography
kgf: Kilogram force
N-m: Newton meter

## Additional Information and Declarations

### Data Availability

The following information was supplied regarding data availability: The questionnaires in the original language (Danish) and English, the dental data and the masticatory cycles and peak bite force values are all available as Supplemental Files.
