# Supplementary material for: Oral function in patients with myasthenia gravis
Source: PeerJ. 2021 Jun 29;9:e11680. doi: 10.7717/peerj.11680 (PMC8253106; doi:10.7717/peerj.11680)
Supplement: Supplemental Information 5 [file peerj-09-11680-s005.pdf]

Odontologiske Landsdels-og Videnscenter
Århus Sygehus

Oral Health Impact Profile (OHIP)

Den oplevede sundhedstilstand i mundhulen og dennes indflydelse på livskvalitet.

Spørgeskema til voksne personer (15 år og ældre).

Formålet med dette spørgeskema er på en standardiseret måde at få beskrevet din egen opfattelse af sundhedstilstanden for dine tænder, mund og kæber samt betydning for livskvalitet.

Spørgsmålene vedrører derfor tænder, mund og kæbers funktion samt spørgsmål om tænder, mund og kæbers betydning for dit velbefindende og dine sociale relationer.

Spørgsmålene er formuleret på en sådan måde, at du skal angive hvor ofte et givet problem er optrådt inden for den sidste måned.

Spørgsmålene bevares med et kryds til hvert spørgsmål. Er der spørgsmål du ikke kan/vil besvare, springes disse over (for eksempel spørgsmål om proteser, hvis du ikke har sådanne)

Spørgeskemaet indgår som en del af din journal.

Navn:

CPR:

Dato:

Marker med et kryds hvor ofte du inden for den seneste måned har oplevet det nævnte problem.

1. **Har du haft vanskeligheder med at tygge nogen form for mad på grund af problemer med dine tænder, mund eller proteser.**

   meget tit temmelig tit af og til næsten aldrig aldrig ved ikke

2. **Har du haft besvær med at udtale visse ord pga. problemer med dine tænder, mund eller proteser.**

   meget tit temmelig tit af og til næsten aldrig aldrig ved ikke

3. **Har du bemærket, om en af dine tænder ikke ser rigtig ud.**

   meget tit temmelig tit af og til næsten aldrig aldrig ved ikke

4. **Har du følt, at dit udseende har været påvirket af problemer med dine tænder, mund eller proteser.**

   meget tit temmelig tit af og til næsten aldrig aldrig ved ikke

5. **Har du følt, at du har haft dårlig ånde pga. problemer med dine tænder, mund eller protese.**

   meget tit temmelig tit af og til næsten aldrig aldrig ved ikke

6. **Har du følt, at din smagssans er blevet forværret pga. problemer med dine tænder, mund eller proteser.**

   meget tit temmelig tit af og til næsten aldrig aldrig ved ikke

7. **Har du haft mad til at sidde fast i tænder eller proteser.**

   meget tit temmelig tit af og til næsten aldrig aldrig ved ikke

8. **Har du følt, at din fordøjelse er blevet forværret pga. problemer med dine tænder, mund eller proteser.**

   meget tit temmelig tit af og til næsten aldrig aldrig ved ikke

9. **Har du følt, at dine proteser ikke har passet ordentlig.**

   meget tit temmelig tit af og til næsten aldrig aldrig ved ikke

10. Har du haft smerter i munden.

meget tit   temmelig tit   af og til   næsten aldrig   aldrig   ved ikke

11. Har du haft ømhed i kæben

meget tit   temmelig tit   af og til   næsten aldrig   aldrig   ved ikke

12. Har du haft hovedpine pga. problemer med dine tænder, mund eller protese.

meget tit   temmelig tit   af og til   næsten aldrig   aldrig   ved ikke

13. Har du haft følsomme tænder, f.eks. ved indtagelse af varm eller kold mad eller drikke.

meget tit   temmelig tit   af og til   næsten aldrig   aldrig   ved ikke

14. Har du haft tandpine.

meget tit   temmelig tit   af og til   næsten aldrig   aldrig   ved ikke

15. Har du haft smerter i gummerne.

meget tit   temmelig tit   af og til   næsten aldrig   aldrig   ved ikke

16. Har du oplevet ubehag ved at spise nogen form for mad pga. problemer med dine tænder, mund eller proteser.

meget tit   temmelig tit   af og til   næsten aldrig   aldrig   ved ikke

17. Har du haft ømme steder i munden

meget tit   temmelig tit   af og til   næsten aldrig   aldrig   ved ikke

18. Har du haft proteser, der sidder dårligt.

meget tit   temmelig tit   af og til   næsten aldrig   aldrig   ved ikke

19. Har du haft bekymringer pga. problemer med tænderne.

meget tit   temmelig tit   af og til   næsten aldrig   aldrig   ved ikke

20. Har du været flov over dine tænder, mund eller proteser.

meget tit   temmelig tit   af og til   næsten aldrig   aldrig   ved ikke

21. Har tandproblemer gjort dig ked af det.

meget tit temmelig tit af og til næsten aldrig aldrig ved ikke

22. Har du følt dig dårligt tilpas pga. udseendet af dine tænder, mund eller proteser.

meget tit temmelig tit af og til næsten aldrig aldrig ved ikke

23. Har du følt dig anspændt pga. problemer med dine tænder, mund eller proteser.

meget tit temmelig tit af og til næsten aldrig aldrig ved ikke

24. Har din taleevne været påvirket pga. problemer med tænder, mund eller proteser.

meget tit temmelig tit af og til næsten aldrig aldrig ved ikke

25. Har folk misforstået nogle af dine ord pga. problemer med dine tænder, mund eller protese.

meget tit temmelig tit af og til næsten aldrig aldrig ved ikke

26. Har du følt, at din mad har smagt af mindre pga. problemer med dine tænder, mund eller proteser.

meget tit temmelig tit af og til næsten aldrig aldrig ved ikke

27. Har du ikke kunnet børste dine tænder ordentlig pga. problemer med dine tænder, mund eller proteser.

meget tit temmelig tit af og til næsten aldrig aldrig ved ikke

28. Har du været nødt til at undgå visse former for mad pga. problemer med dine tænder, mund eller proteser.

meget tit temmelig tit af og til næsten aldrig aldrig ved ikke

29. Har din kost været utilfredsstillende pga. problemer med dine tænder, mund eller protese.

meget tit temmelig tit af og til næsten aldrig aldrig ved ikke

30. Har du været ude af stand til at spise pga. problemer med dine proteser.

meget tit temmelig tit af og til næsten aldrig aldrig ved ikke

31. Har du undgået at smile pga. problemer med dine tænder, mund eller proteser.

meget tit temmelig tit af og til næsten aldrig aldrig ved ikke

32. Har du måttet afbryde måltider pga. problemer med dine tænder, mund eller proteser.

meget tit temmelig tit af og til næsten aldrig aldrig ved ikke

33. Er din søvn blevet forstyrret pga. problemer med dine tænder, mund eller proteser

meget tit temmelig tit af og til næsten aldrig aldrig ved ikke

34. Har du været ud af balance pga. problemer med dine tænder, mund eller proteser.

meget tit temmelig tit af og til næsten aldrig aldrig ved ikke

35. Har du haft svært ved at slappe af pga. problemer med dine tænder, mund eller proteser.

meget tit temmelig tit af og til næsten aldrig aldrig ved ikke

36. Har du været nedtrykt pga. problemer med dine tænder, mund eller proteser.

meget tit temmelig tit af og til næsten aldrig aldrig ved ikke

37. Har din koncentrationsevne været påvirket pga. af problemer med dine tænder, mund eller proteser.

meget tit temmelig tit af og til næsten aldrig aldrig ved ikke

38. Har du været en smule forlegen pga. problemer med dine tænder, mund eller proteser.

meget tit temmelig tit af og til næsten aldrig aldrig ved ikke

39. Har du undladt at gå ud pga. problemer med dine tænder, mund eller proteser.

meget tit temmelig tit af og til næsten aldrig aldrig ved ikke

40. Har du været mindre tolerant over for din ægtefælle eller familie pga. problemer med dine tænder, mund eller proteser.

meget tit temmelig tit af og til næsten aldrig aldrig ved ikke

41. Har du haft problemer med at omgås andre mennesker pga. problemer med dine tænder, mund eller proteser.

meget tit temmelig tit af og til næsten aldrig aldrig ved ikke

42. Har du været lidt irritabel over for andre mennesker pga. problemer med dine tænder, mund eller proteser.

meget tit temmelig tit af og til næsten aldrig aldrig ved ikke

43. Har du haft besvær med at passe dit sædvanlige arbejde pga. problemer med dine tænder, mund eller proteser.

meget tit | temmelig tit | af og til | næsten aldrig | aldrig | ved ikke

44. Har du følt, at dit helbred er blevet forværret på grund af problemer med dine tænder, mund eller proteser.

meget tit | temmelig tit | af og til | næsten aldrig | aldrig | ved ikke

45. **Har du lidt nogen form for økonomisk tab pga. problemer med dine tænder, mund eller proteser**.

meget tit | temmelig tit | af og til | næsten aldrig | aldrig | ved ikke

46. Har du været ude af stand til at nyde andre menneskers selskab pga. problemer med dine tænder, mund eller proteser.

meget tit | temmelig tit | af og til | næsten aldrig | aldrig | ved ikke

47. Har du følt, at livet i almindelighed var mindre tilfredsstillende pga. problemer med dine tænder, mund eller proteser

meget tit | temmelig tit | af og til | næsten aldrig | aldrig | ved ikke

48. Har du været helt ude af stand til at fungere pga. problemer med dine tænder, mund eller proteser.

meget tit | temmelig tit | af og til | næsten aldrig | aldrig | ved ikke

49. Har du været ude af stand til at arbejde på fuld kraft pga. problemer med dine tænder, mund eller proteser.

meget tit | temmelig tit | af og til | næsten aldrig | aldrig | ved ikke
