# Supplementary material for: Oral function in patients with myasthenia gravis
Source: PeerJ. 2021 Jun 29;9:e11680. doi: 10.7717/peerj.11680 (PMC8253106; doi:10.7717/peerj.11680)
Supplement: Supplemental Information 6 [file peerj-09-11680-s006.pdf]

Table 1. The 49 items of the Oral Health Impact Profile (OHIP) organized into 7 subscales

Functional limitation

- Have you had difficulty chewing any foods because of problems with your teeth, mouth or dentures?
- Have you had trouble pronouncing any words because of problems with your teeth, mouth or dentures?
- Have you noticed a tooth which doesn't look right?
- Have you felt that your appearance has been affected because of problems with your teeth, mouth or dentures?
- Have you felt that your breath has been stale because of problems with your teeth, mouth or dentures?
- Have you felt that your sense of taste has worsened because of problems with your teeth, mouth or dentures?
- Have you had food catching in your teeth or dentures?
- Have you felt that your digestion has worsened because of problems with your teeth, mouth or dentures?
- Have you felt that your dentures have not been fitting properly?

Physical pain

- Have you had painful aching in your mouth?
- Have you had a sore jaw?
- Have you had headaches because of problems with your teeth, mouth or dentures?
- Have you had sensitive teeth, for example, due to hot or cold foods or drinks?
- Have you had toothache?
- Have you had painful gums?
- Have you found it uncomfortable to eat any foods because of problems with your teeth, mouth or dentures?
- Have you had sore spots in your mouth?
- Have you had uncomfortable dentures?
- Psychological discomfort
- Have you been worried by dental problems?
- Have you been self conscious because of your teeth, mouth or dentures?
- Have dental problems made you miserable?
- Have you felt uncomfortable about the appearance of your teeth, mouth or dentures?
- Have you felt tense because of problems with your teeth, mouth or dentures?

Physical disability

- Has your speech been unclear because of problems with your teeth, mouth or dentures?
- Have people misunderstood some of your words because of problems with your teeth, mouth or dentures?
- Have you felt that there has been less flavour in your food because of problems with your teeth, mouth or dentures?
- Have you been unable to brush your teeth properly because of problems with your teeth, mouth or dentures?
- Have you had to avoid eating some foods because of problems with your teeth, mouth or dentures?
- Has your diet been unsatisfactory because of problems with your teeth, mouth or dentures?
- Have you been unable to eat with your dentures because of problems with them?
- Have you avoided smiling because of problems with your teeth, mouth or dentures?
- Have you had to interrupt meals because of problems with your teeth, mouth or dentures?

Psychological disability

- Has your sleep been interrupted because of problems with your teeth, mouth or dentures?
- Have you been upset because of problems with your teeth, mouth or dentures?
- Have you found it difficult to relax because of problems with your teeth, mouth or dentures?
- Have you felt depressed because of problems with your teeth, mouth or dentures?
- Has your concentration been affected because of problems with your teeth, mouth or dentures?
- Have you been a bit embarrassed because of problems with your teeth, mouth or dentures?

Social disability

- Have you avoided going out because of problems with your teeth, mouth or dentures?
- Have you been less tolerant of your spouse or family because of problems with your teeth, mouth or dentures?
- Have you had trouble getting on with other people because of problems with your teeth, mouth or dentures?
- Have you been a bit irritable with other people because of problems with your teeth, mouth or dentures?
- Have you had difficulty doing your usual jobs because of problems with your teeth, mouth or dentures?

Handicap

- Have you felt that your general health has worsened because of problems with your teeth, mouth or dentures?
- Have you suffered any financial loss because of problems with your teeth, mouth or dentures?
- Have you been unable to enjoy other people's company as much because of problems with your teeth, mouth or dentures?
- Have you felt that life in general was less satisfying because of problems with your teeth, mouth or dentures?
- Have you been totally unable to function because of problems with your teeth, mouth or dentures?
- Have you been unable to work to your full capacity because of problems with your teeth, mouth or dentures?
